# Preliminary embryo-fetal developmental toxicity study of pritelivir in CD1-IGS mice

**DOI:** 10.1038/s41598-026-57669-x

**Published:** 2026-06-21

**Authors:** Tatiana N. Nanovskaya, Svetlana L. Patrikeeva, Xiaoming Wang, Erik Rytting, Bandana Bera, Sydni C. Zacharias, Brendan Prideaux, Valentina F. Bryant

**Affiliations:** 1 Maternal-Fetal Pharmacology and Bio-Development Laboratories, Department of Obstetrics & Gynecology, The University of Texas Medical Branch, 301 University Boulevard, Galveston, TX 77555-0587 USA; 2 Department of Neurobiology, The University of Texas Medical Branch, 301 University Boulevard, Galveston, TX 77555-0620 USA

**Keywords:** Pritelivir, Preliminary embryo-fetal developmental toxicity study, Toxicokinetic, Fetoplacental compartment, Diseases, Medical research

## Abstract

Pritelivir is a novel anti-herpes simplex virus drug that is currently in clinical trials in nonpregnant immunocompromised subjects. Here we conducted a preliminary embryo-fetal developmental toxicity study of pritelivir in CD1-IGS mice. Pritelivir was administered by oral gavage to time-mated mice at dose levels of 0, 10, 60 and 200 mg/kg/day from gestational day 6 through 15. Maternal food consumption, body weight and clinical observations were monitored throughout gestation. The mice were sacrificed at gestational day 18 and assessed based on embryo-fetal development and viability. Fetuses were weighed and examined for external and visceral variations and malformations. The results showed that exposure to pritelivir did not cause maternal or developmental toxicity in mice. Thus, both maternal and fetal no-observed-adverse-effect-level of pritelivir was determined to be 200 mg/kg/day. At this dose, the area under the concentration-time curve for pritelivir at steady state in maternal animals was 20 times greater than reported in humans at the clinical dose of 100 mg/day. Placenta-to-maternal plasma and fetus-to-maternal plasma concentration ratios in mice were 0.4 and 0.2, respectively, indicating transfer but not accumulation of pritelivir in the fetoplacental compartment. Taken together, our findings support further preclinical studies of pritelivir for its use in pregnancy.

**Supplementary Information:**

The online version contains supplementary material available at https://doi.org/10.1038/s41598-026-57669-x.

## Introduction

Genital herpes is caused by Herpes simplex viruses (HSV) types 1/2 and affects 1 out of 6 people of 14–49 years of age, which corresponds to the age of childbearing potential in women^1^. In pregnancy, primary and recurrent episodes of genital herpes present the risk of mother-to-neonate transmission of the virus^2^. Neonatal HSV infection has an estimated incidence rate ranging from 1/20,000 to 1/3,000 live births and can be life threatening^3^. While the nucleoside analog acyclovir and its pro-drug, valacyclovir, can be used during pregnancy in patients with genital HSV to reduce the frequency and severity of disease outbreaks, they do not prevent subclinical shedding of the virus^4,5^, therefore, the HSV transmission to the neonates is still possible at vaginal birth^6^. Moreover, emerging acyclovir resistance in HSV represents a clinical problem, particularly among immunocompromised patients. While pregnant women are not considered immunocompromised^7^, it is not known whether pregnancy-associated immunologic changes increase the risk of the development of acyclovir resistance. On the other hand, women who are immunosuppressed (e.g., organ transplants recipients and patients with autoimmune diseases) can get pregnant and carry pregnancy to term^8^. Foscarnet (pyrophosphate analog) and cidofovir (nucleotide analog) are typically used in the treatment of acyclovir-resistant HSV; however, their use in pregnancy is not recommended due to their potential nephrotoxicity and reported teratogenicity in animal reproductive toxicity studies^9,10^.

**Fig. 1:**
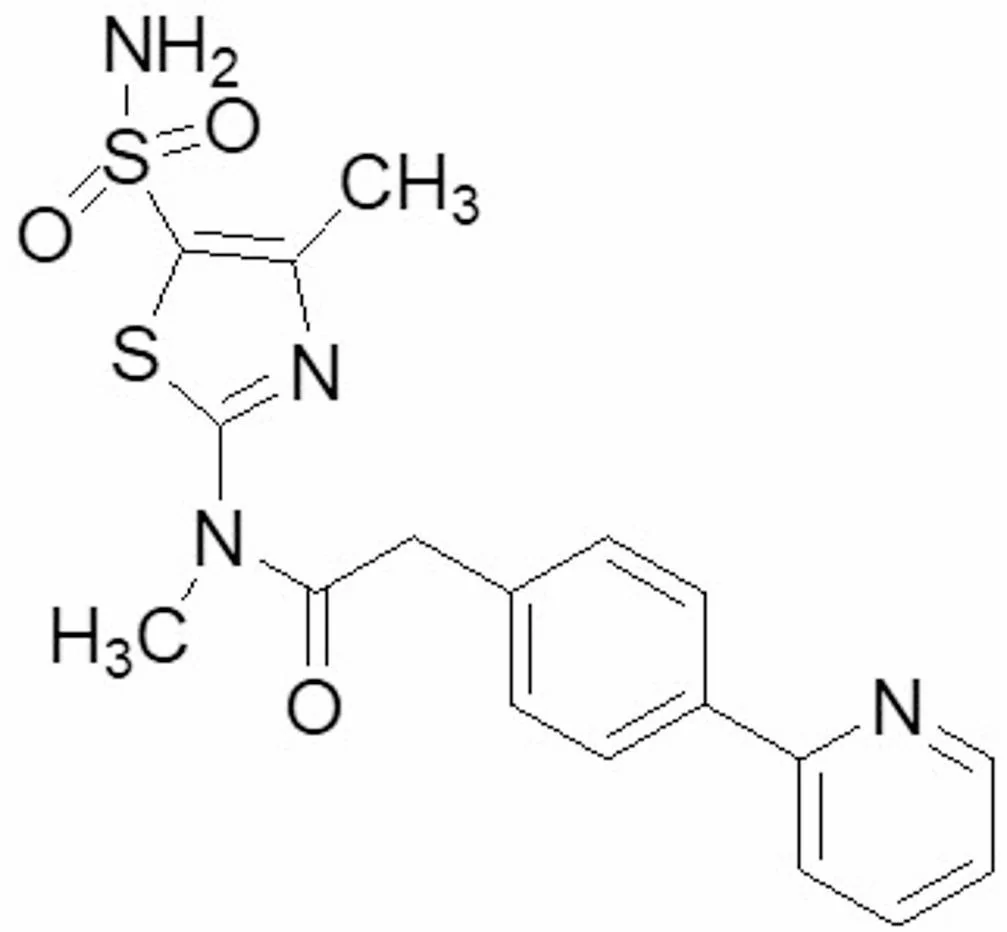
Pritelivir structure.

Pritelivir, a helicase-primase inhibitor, is a novel anti-HSV agent that was developed to circumvent the limitations of the nucleoside analogs and is currently in clinical development (Fig. 1). A recent phase 3 clinical trial assessed the drug’s safety and efficacy in the treatment of acyclovir-resistant mucocutaneous HSV infections in immunocompromised subjects [ClinicalTrials.gov ID: NCT03073967] with encouraging results (control study arm received either foscarnet or cidofovir)^11^. Pritelivir may also be available to eligible immunocompromised individuals as part of the Expanded Access Program [ClinicalTrials.gov ID: NCT05844436]. Pregnant subjects are not eligible to participate in these studies, and participants who can get pregnant are required to use highly effective birth control methods with < 1% failure rate. To the best of our knowledge, developmental toxicity and viability data for pritelivir have not been reported previously.

As part of the preclinical development of pritelivir for its use in pregnancy, we recently conducted an ex vivo perfusion of this drug across term human placental lobules at the concentration of 5.5 µg/mL, which was within the range of the reported maximum plasma concentration (C_max_) for the dose utilized in ongoing clinical trials^12^. Our results revealed that pritelivir crosses human placental tissue indicating possible fetal exposure to the drug^12^. The drug did not alter the viability or functionality of the perfused placental lobule ex vivo at the estimated C_max_, and did not illicit cytotoxicity in human choriocarcinoma-derived trophoblast cells (BeWo, b30 clone) that are a commonly used in vitro experimental model for human placental biology, and human umbilical vein endothelial cells (HUVEC) in vitro at concentrations exceeding the estimated C_max_ by six-fold^12^. Together, the results from our ex vivo and in vitro studies suggest favorable safety profile of pritelivir in the placental tissue. This publication describes the findings of our preliminary embryo-fetal developmental (pEFD) toxicity study and a satellite toxicokinetic study of pritelivir in mice, aiming to further inform on the development of this novel anti-HSV drug for its use in pregnancy.

## Results

### Maternal effects and litter data

**Fig. 2:**
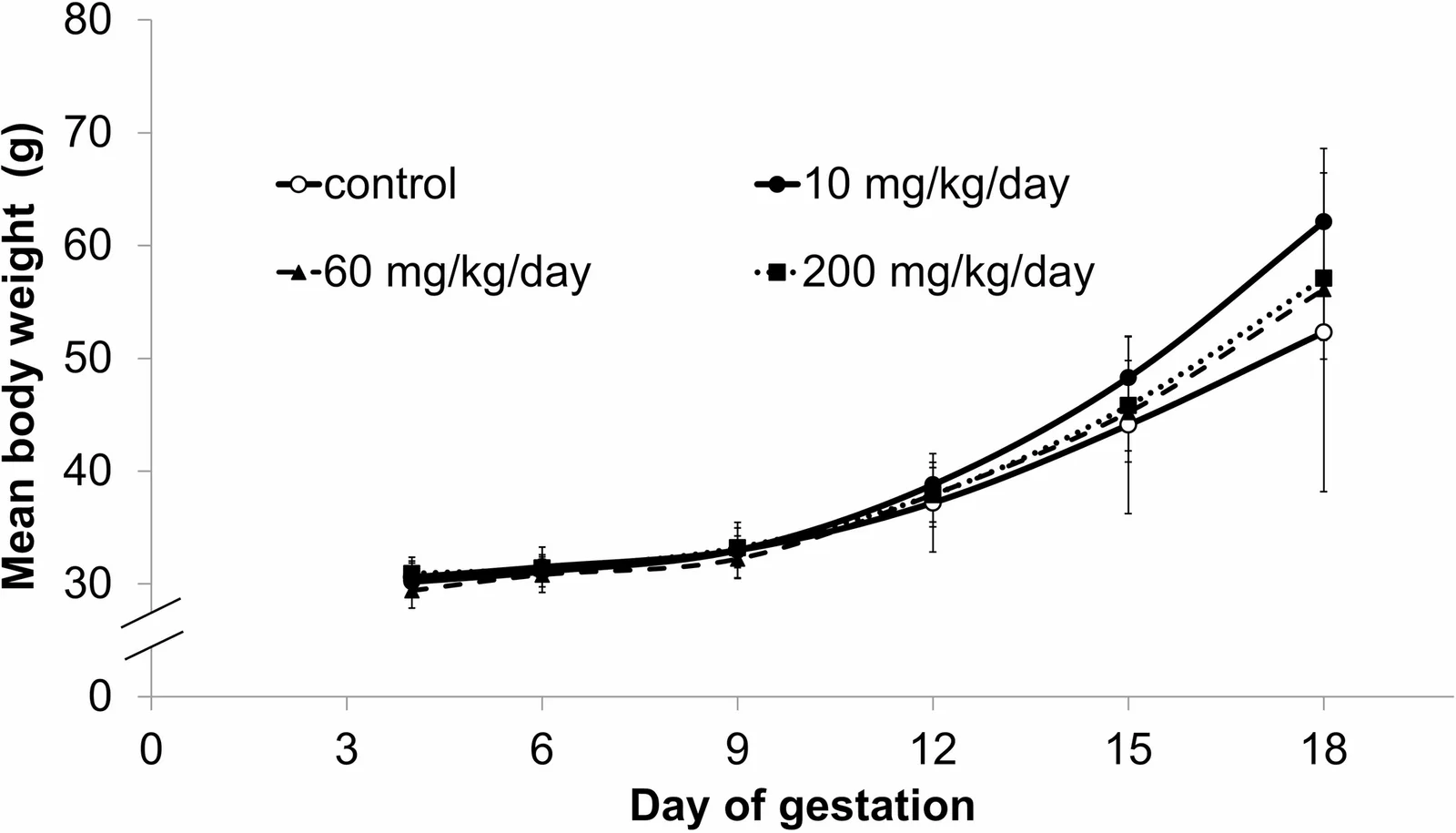
Mean weight of pregnant mice from the pEFD study of pritelivir administered via oral gavage from GD 6 through 15. Body weights at GD 4 were similar across the study groups and were as follows: control, 30.6 ± 1.8 g (*n* = 9); low dose exposure, 30.2 ± 1.8 g (*n* = 8), middle dose, 29.4 ± 1.5 g (*n* = 6) and high dose, 30.9 ± 1.0 g (*n* = 9). Data presented as mean ± S.D.

Clinical observations did not reveal any pritelivir-related effects, and no unscheduled deaths occurred in either treatment group of 0 (vehicle control), 10, 60 and 200 mg/kg/day. As mentioned in the Materials and Methods section for the pEFD study, one animal from the control group was found with fetal uterine contents and fetuses in the cage on gestational day (GD) 18 prior to the scheduled sacrifice. There was no statistically significant treatment-related effect on mean body weight or mean weight gain of dams. Notably, the body weight was increased in pritelivir-treated mice as compared to the control group, and while the effect was not dose-dependent and lacked statistical significance, it was considered potentially pritelivir-related (Fig. 2, Tables S1&S2). No pritelivir-related effect on mean food consumption was observed.

One control animal had no viable fetuses at the time of necropsy. It must be noted that implantation in mice takes place at GD 4^13^, which was prior to dosing in the current study; therefore, the lack of pregnancy in any animals from any group in this study was deemed not to be vehicle or pritelivir related. In addition, *n* = 8 time-mated animals were allocated per treatment group originally to obtain at least *n* = 6 pregnant animals at the end of the study as recommended for the pEFD study design^14–16^. However, due to insufficient number of pregnant animals that were randomly assigned to the high dose pritelivir-treated group originally, additional time-mated mice were ordered to ensure that each group had at least *n* = 6 pregnant animals at the time of sacrifice. Thus, the total of forty time-mated female CD1-IGS mice were used in our pEFD study, and the resulting pregnancy rates were 90, 100, 75 and 64.3% for animals administered vehicle, low, medium and high doses of pritelivir, respectively. The observed pregnancy rates were comparable to the historical control data reported by Charles River Laboratories (the supplier of laboratory animals for our study) for in-house bred and time-mated CD1-IGS mice^12,17,18^.

No gravid uterine weight or placental weights were recorded for the dam from the control group that delivered prior to sacrifice at GD 18 (mentioned earlier). Eight live fetuses and one cannibalized fetus that were found in the cage were arbitrarily assigned to the implantation sites. Mean numbers of corpora lutea, implantation sites, preimplantation losses, and post implantation losses (early resorptions, late resorptions and dead fetuses) were comparable among the groups, and no difference was noted in the mean gravid uterine weight, mean placental weight, mean fetal weight (male, female or combined). Likewise, no treatment-related macroscopic changes were noted, and histopathological examination of uteri, cervix, urinary bladder and placental tissues did not reveal any treatment-related changes. Maternal effects as well as summary of cesarean section data and mean fetal data are also summarized in Tables S3&S4. The number of viable fetuses per litter was similar in all treatment groups in our pEFD study and was consistent with the historical control values reported by Charles River Laboratories for in-house bred and time-mated CD1-IGS mice^12,17,18^.

### Fetal examination

The summary of fetal external and visceral observations and malformations are presented in Tables 1 and 2. All external malformations and visceral malformations and variations were deemed as single incidences and were observed in the vehicle-treated group only or were not dose-dependent in the pritelivir-treated groups. Among the observed visceral malformations, small kidneys were revealed in three fetuses in one animal from the 10 mg/kg/day (i.e., low dose) treated group. However, no additional fetuses showed this malformation in either group, therefore this malformation was considered to be a litter-effect and was deemed not related to the exposure to pritelivir. The incidence of fetal visceral anomalies observed in CD1-IGS mice in our study was consistent with the historical control data from the testing facility LabCorp Early Development Laboratories, Inc (Table S5) as well as those reported by Charles River Laboratories^12,17,18^. As a result, both maternal and fetal no-observed-adverse-effect-level (NOAEL) of pritelivir was determined to be 200 mg/kg/day, which was the highest dose of the drug in our study.

**Table 1:** Summary of fetal external malformations.

	Pritelivir dose, mg/kg/day	0	10	60	200
Tissue observation	Number of maternal females/fetuses	10/90	8/105	8/71	14/112
	Number of examined litters/fetuses	8/90	8/105	6/71	9/112
Eye – open	Number of affected litters/fetuses	0/0	1/1^b^	0/0	0/0
	Proportion of litter with observation (%)	0%	13%	0%	0%
	Mean incidence^a^ (% per litter)	0%	1.04%	0%	0%
Head –exencephale	Number of affected litters/fetuses	0/0	1/1^b^	0/0	0/0
	Proportion of litter with observation (%)	0%	13%	0%	0%
	Mean incidence^a^ (% per litter)	0%	1.04%	0%	0%
General - omphalocele	Number of affected litters/fetuses	0/0	1/1	0/0	0/0
	Proportion of litter with observation (%)	0%	13%	0%	0%
	Mean incidence^a^ (% per litter)	0%	0.89%	0%	0%
Limb - malrotated	Number of affected litters/fetuses	1/1	0/0	0/0	0/0
	Proportion of litter with observation (%)	13%	0%	0%	0%
	Mean incidence^a^ (% per litter)	1.14%	0%	0%	0%

Proportion of litter with observation (%) were compared using Fisher exact test (upper tail).

% per litter values were not analyzed statistically (to too few distinct values).

^a^Mean incidence (% per litter) = average percentage of affected fetuses per litter in each group.

^b^Both malformations observed on the same fetus.

**Table 2:** Summary of fetal visceral malformations and variations.

Tissue observation		Pritelivir, mg/kg/day	0	10	60	200
		Number of maternal females/fetuses	10/90	8/105	8/71	14/112
		Description	Classification	Number of examined litters/fetuses	8/90	8/105
Kidney - small	Malformation	Number of affected litters/fetuses	0/0	1/3	0/0	0/0
		Proportion of litter with observation (%)	0%	13%	0%	0%
		Mean incidence^a^ (% per litter)	0%	3.13%	0%	0%
Blood vessel – umbilical artery left-sided	Variation	Number of affected litters/fetuses	1/2	4/6	2/2	3/7
		Proportion of litter with observation (%)	13%	50%	33%	33%
		Mean incidence^a^ (% per litter)^b^	2.27%	5.94%	2.67%	5.77%
Kidney-misshapen	Variation	Number of affected litters/fetuses	0/0	0/0	1/1	0/0
		Proportion of litter with observation (%)	0%	13%	17%	0%
		Mean incidence^a^ (% per litter)	0%	0.89%	1.28%	0%
Lung – intermediate/accessory lobe absent	Variation	Number of affected litters/fetuses	1/1	0/0	0/0	0/0
		Proportion of litter with observation (%)	13%	0%	0%	0%
		Mean incidence^a^ (% per litter)	2.08%	0%	0%	0%
Spleen - discolored	Variation	Number of affected litters/fetuses	0/0	1/2	1/1	0/0
		Proportion of litter with observation (%)	0%	13%	17%	0%
		Mean incidence^a^ (% per litter)	0%	1.92%	1.11%	0%

Proportion of litter with observation (%) were compared using Fisher exact test (upper tail).

% per litter values were not analyzed statistically (to too few distinct values).

^a^Mean incidence (% per litter) = average percentage of affected fetuses per litter in each group.

^b^% of fetal values for this parameter were compared using Kruskal-Wallis and Wilcoxon Sum Rank test.

### Toxicokinetic study

**Fig. 3:**
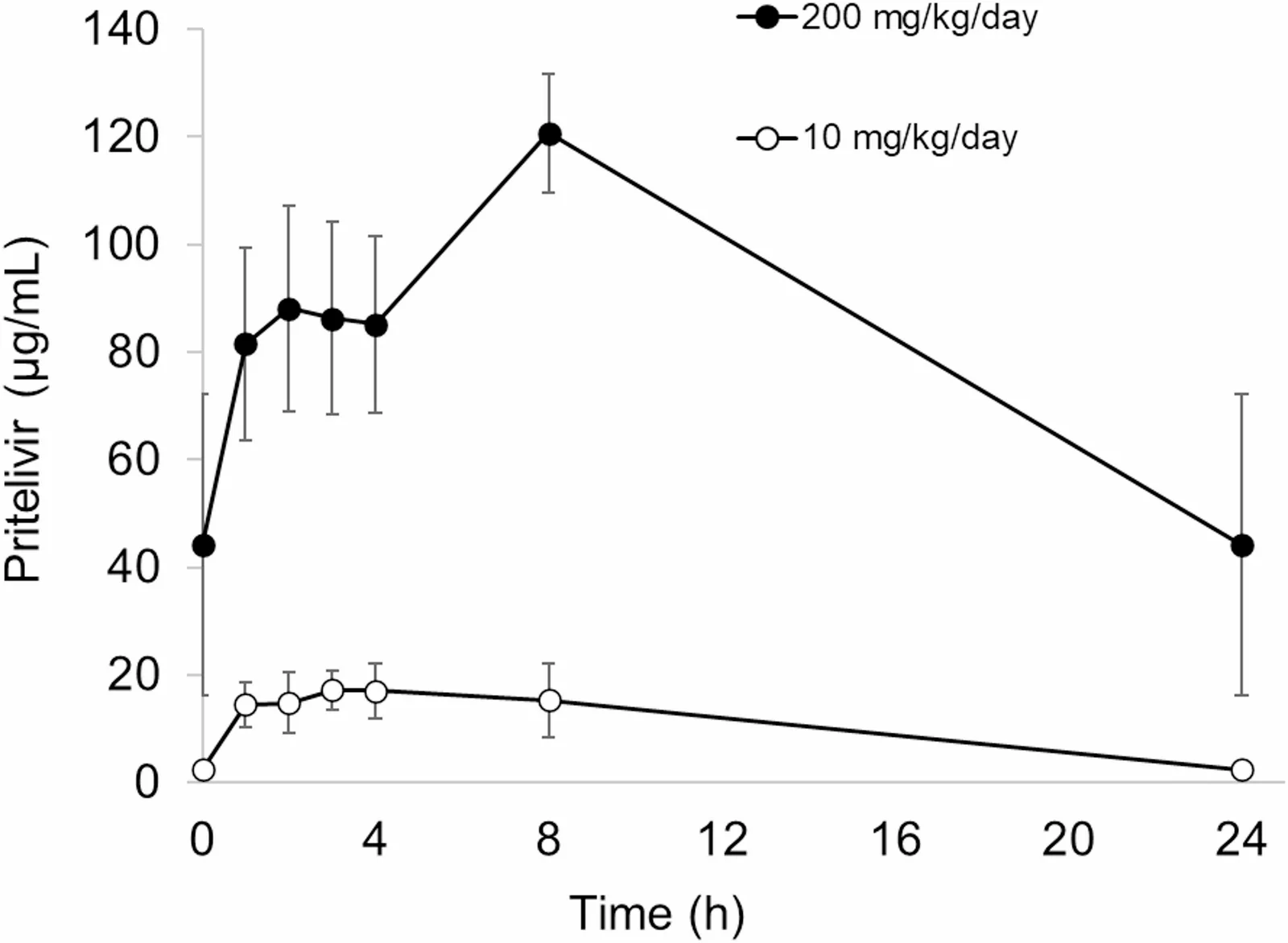
Plasma concentration-time profiles for pritelivir administered to pregnant CD1-IGS mice via gavage at dose levels of 10 and 200 mg/kg/day from gestational day 9 through 13. Animals were sacrificed by CO_2_ asphyxiation, followed by bilateral thoracotomy, at the predetermined time points of 1, 2, 3, 4, 8, and 24 h post-dose, and blood samples were obtained by cardiac puncture. Pritelivir in plasma samples was quantified using LC-MS/MS. Data are presented as the mean ± S.D. of three separate experiments that utilized 1–3 animals per time point.

The pharmacokinetic (PK) profiles of pritelivir in the plasma of pregnant as well as nonpregnant mice following repeated doses of 10 mg/kg/day and 200 kg/mg/day of oral pritelivir are shown in Figs. 3 & S1, respectively, and the PK parameters are recorded in Table S6. Accordingly, the area under the concentration-time curve at steady state (AUC_ss_) and C_max_ values for pritelivir in maternal animals at the NOAEL dose (200 mg/kg/day) indicated a 20-fold margin over anticipated human exposure at clinical dosing of 100 mg/day of pritelivir based on the reported plasma AUC_ss_ and C_max_ values for this drug (102.9 ± 25.2 µg×h/mL and 5.9 ± 1.9 µg/mL, respectively) in non-pregnant human subjects^19^. The plasma exposure in pregnant mice at the 10 mg/kg/day dose of pritelivir resembled human exposure at clinical dosing more closely and was 2.6- and 3.1-fold higher than the reported plasma AUC_ss_ and C_max_ values in humans^19^. The exposure between the 10 mg/kg/day and 200 mg/kg/day doses was not dose proportional, with the exposure margins at 10 mg/kg/day were 7.9- and 6.5-fold higher for AUC_ss_ and C_max_, respectively.

It appears that pregnancy did not affect the AUC_ss_ and C_max_ values of pritelivir in mice at both studied doses at steady state (Table S6). Furthermore, the PK profile of pritelivir at the dose of 10 mg/kg/day was similar in pregnant (Fig. 3) and nonpregnant female mice (Figure S1). On the other hand, a split-peak PK profile was revealed in the plasma of pregnant mice treated with 200 mg/kg/day (Fig. 3) with two distinctive peaks at 2.3 ± 0.6 h and 8 h, with the second peak representing the C_max_, while monophasic kinetics was observed in nonpregnant female mice at this dose (Figure S1). Distribution of pritelivir in the tissues were similar in pregnant and nonpregnant female mice treated with either dose of the drug (Table S7&S8) and was the most extensive in the liver (tissue AUC_ss_/plasma AUC_ss_ ratios were 1.17 ± 0.77 and 1.16 ± 0.25 in pregnant and 0.66 ± 0.13 and 1.07 ± 0.15 in nonpregnant female mice treated with 10 mg/kg/day and 200 mg/kg/day dose, respectively). Meanwhile, the lowest exposure to pritelivir was revealed in the brain, for which tissue AUC_ss_/plasma AUC_ss_ ratios were 0.30 ± 0.18 and 0.25 ± 0.13 for pregnant, and 0.23 ± 0.07 and 0.24 ± 0.05 for nonpregnant mice treated with low and high dose of pritelivir, respectively.

Table 3 shows the PK parameters of pritelivir for the placental and fetal tissues and the concentrations of pritelivir in the amniotic fluid at the plasma T_max_. Pritelivir AUC_ss_ and C_max_ in the placental and fetal tissues as well as concentrations of the drug in the amniotic fluid at the maternal dose of 200 mg/kg/day were higher than at the dose of 10 mg/kg/day. The placenta AUC_ss_ /plasma AUC_ss_ and fetus AUC_ss_ /plasma AUC_ss_ ratios of the drug as well as the respective concentration ratios at T_max_ appeared similar irrespective of the dose (Table 3). No accumulation of pritelivir was observed in the amniotic fluid.

**Table 3:** Pharmacokinetic parameters of pritelivir in the maternal plasma, fetuses and placental tissues of pregnant CD1-IGS mice after oral administration of multiple doses of 10 mg/kg/day and 200 mg/kg/day.

Tissue	Maternal dose (mg/kg/day)	C_max_ (µg/mL or µg/g)^a^	AUC_ss_ (μg×h/mL or µg×h/g)^a^	T_max_ (h)	Ratio of tissue/maternal plasma concentrations at plasma T_max_^c^	Ratio of tissue/maternal plasma concentrations at tissue T_max_	Ratio of tissue/plasma AUC_ss_ values
Maternal plasma	10	18.5 ± 4.4	262 ± 103	4.3 ± 3.2	NA	NA	NA
Placenta	10	7.89 ± 0.88	108 ± 7	3.0 ± 0.0	0.39 ± 0.14	0.47 ± 0.07	0.46 ± 0.18
Fetus	10	3.32 ± 0.49	48.0 ± 6.2	3.0 ± 1.0	0.18 ± 0.06	0.19 ± 0.05	0.20 ± 0.07
Amniotic fluid^b^	10	0.97 ± 0.17^b^	ND	ND	0.05 ± 0.01^c^	ND	ND
Maternal plasma	200	121 ± 11.1	2057 ± 285	8.0 ± 0.0	NA	NA	NA
Placenta	200	58.8 ± 15.3	955 ± 293	4.7 ± 3.1	0.43 ± 0.10	0.58 ± 0.12	0.46 ± 0.09
Fetus	200	27.5 ± 4.9	445 ± 127	4.7 ± 3.1	0.20 ± 0.05	0.28 ± 0.05	0.22 ± 0.04
Amniotic fluid^b^	200	3.20 ± 0.45^b^	ND	ND	0.029 ± 0.004^c^	ND	ND

Data presented as mean ± S.D. of three experiments, each point represented by one to three maternal animals for maternal plasma and placental and fetal tissues. For the amniotic fluid, data presented as mean ± S.D of *n* = 4 samples collected from individual amniotic sacs of one maternal animal per treatment group.

^a^The units are µg/mL for C_max_ and μg×h/mL for AUC_ss_ for maternal plasma and amniotic fluid, and µg/g and μg×h/g for the respective PK parameters for placental and fetal tissues.

^b^Data presented as mean ± S.D. of *n* = 4 samples obtained from one pregnant mouse at anticipated plasma T_max_ for each dose of pritelivir.

^c^The ratio of amniotic fluid/maternal plasma concentration was calculated using pritelivir plasma concentration from the corresponding maternal animal and presented as mean ± S.D.

NA, not applicable; ND, not determined.

### Desorption electrospray ionization mass spectrometry imaging (DESI-MSI) data

**Fig. 4:**
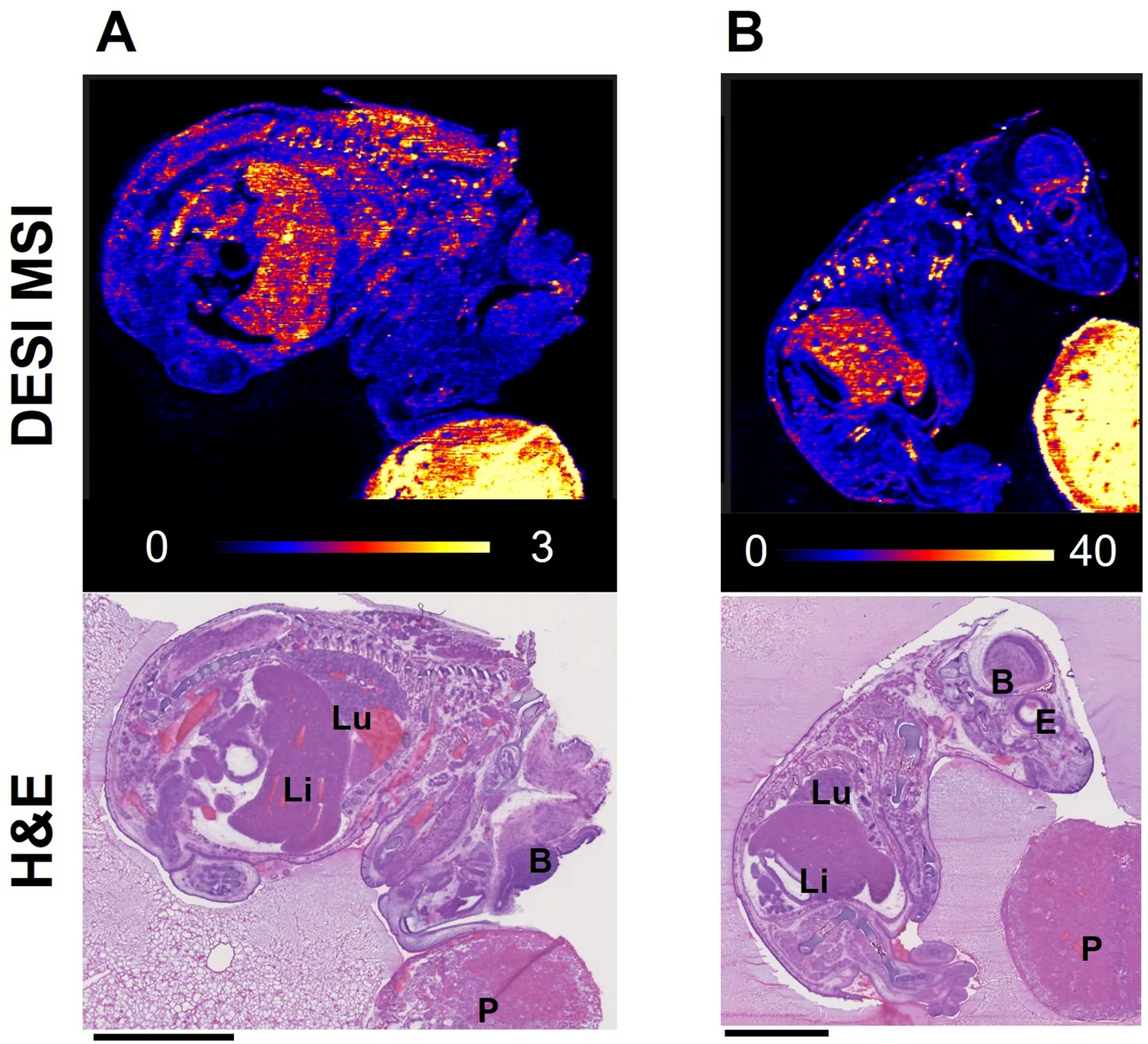
Visualization of pritelivir (m/z 491→196) in CD1-IGS mouse fetal tissues by targeted DESI-MSI following treatment of pregnant mice with 10 mg/kg/day (**A**) and 200 mg/kg/day (**B**) from gestational day 9 through 13. Animals were sacrificed by CO_2_ asphyxiation, followed by bilateral thoracotomy, at 4 h and 8 h postdose 10/mg/kg/day and 200 mg/kg/day, respectively. MSI data were acquired at 40 mm pixel size. DESI-MS signal intensities were normalized to pritelivir-d_4_ internal standard spiked into the DESI spray solvent, and MS images are presented scaled individually for each dose. Scalebar = 4 mm. B, brain; E, eye; Li, liver; Lu, lung; P, placenta; H&E, Hematoxylin and Eosin staining.

The distribution of pritelivir within the placental and fetal tissue of pritelivir-treated pregnant mice was visualized by DESI-MSI analysis corroborating our liquid chromatography-mass spectrometry (LC-MS/MS) findings (Fig. 4&S2). Accordingly, the relative ion signal intensity was higher in the placental tissue than in the corresponding fetal tissues irrespective of the maternal dose of pritelivir (Figure S2D). Within the fetus, the most extensive distribution of the drug was within the liver (Fig. 4&S2D). No notable noise or interfering background signals were detected in the fetal and placental tissues obtained from pregnant mice treated with vehicle (control group) (Figure S2C). High ion signal intensity was observed in the maternal liver irrespective of dose, and the drug was also visualized in the maternal brain (Figure S2A&B). These tissues were included in the DESI-MSI analysis as additional control samples due to their extensive and less-than-extensive respective exposure to the drug in pritelivir-treated mice as revealed by the LC-MS/MS analysis. It appears that within the tested samples of murine brain the highest ion signal intensity for pritelivir was observed in brain ventricles (Figure S2A&B).

## Discussion

HSV infections represent significant public health concern due to high prevalence of the infection in the population and the risk of the transmission during asymptomatic stages. Neonatal HSV infections can cause serious morbidity and mortality, despite advances in diagnosis and availability of the standard of care treatment with intravenous acyclovir for neonates^20^. Up to 85% of neonatal HSV is acquired during vaginal birth from mothers with genital herpes who may or may not have clinically evident symptoms of active disease^21^. The standard of care therapy with acyclovir and valacyclovir has been available to pregnant women for decades. However, it does not abrogate asymptomatic shedding of the virus and is not helpful against emerging acyclovir-resistant infections. Acyclovir-resistant HSV is more common among immunocompromised individuals and is usually treated with foscarnet and cidofovir. However, these drugs are contraindicated in pregnancy, as was mentioned previously.

The helicase-primase inhibitor pritelivir outperformed nucleoside analogs in efficacy and potency in preclinical studies and clinical trials and is effective against acyclovir-resistant HSV strains^17,22^. While ongoing clinical development of pritelivir is focused on immunocompromised patients, expanding the patient population to include other groups such as immunocompetent individuals could be anticipated in the future. Pregnant patients with genital herpes, particularly acyclovir-resistant strains, may benefit from pritelivir to reduce the risk of perinatal transmission of the virus as well as improving the quality of life. Therefore, preclinical reproductive and developmental toxicity studies for pritelivir in vivo constitute an imperative step in the clinical development of this drug for its potential use in pregnancy.

An exploratory 4-week chronic toxicity study of pritelivir in dogs with 30, 100 and 300 mg/kg once daily dosing revealed no relevant safety finding^23^. Therefore, the NOAEL in dogs was considered to be 300 mg/kg, exceeding the dose of 100 mg/day utilized in the clinical development of pritelivir in nonpregnant subjects by 180-fold when converted to the human equivalent dose (HED) based on surface area^24^. However, the same exposure rate and duration in rats resulted in a dose-dependent transitional hyperplasia of the epithelium in the urinary bladder likely due to the inhibitory effect of pritelivir on carbonic anhydrase activity attributed to its aromatic primary sulfonamide pharmacophore^23^. Primary sulfonamide drugs (e.g. acetazolamide) can inhibit carbonic anhydrase enzymes in humans, dogs and rodents leading to hyperplasia in rodents only, but not in other species; therefore, the observed effect of pritelivir in rats was deemed species-specific^23,25^. Kleymann et al.^23^ reported an IC_50_ of 2–5 µM for pritelivir towards carbonic anhydrases in vitro, and the exposure in rats in the chronic toxicity study mentioned above exceeded 100 µM at high doses^23^.

Our pEFD study on mice with pritelivir was designed following the guidelines of regulatory agencies^14,15^ and recommendation of Wise et al.^16^ with respect to the number of animals per treatment group and endpoints of the study. Accordingly, skeletal examinations were not performed, which represent the major limitation of our study (skeletal examinations are part of definitive EFD studies, which also require a larger number of animals^16^. As mentioned above, an earlier chronic toxicity study revealed that pritelivir caused bladder epithelial hyperplasia in rats; the effect was reversible and was deemed rodent-specific^23,25^. Accordingly, we included microscopic examination of urinary bladder of the dams as additional endpoint, as well as histological examination of the placental and uterine tissues.

Exposure to pritelivir did not lead to mortality of maternal animals in our study. Pritelivir demonstrated no significant impact on maternal clinical observations, body weight, food consumption, reproductive performance, anatomic pathology, and cesarean section parameters. Our study also revealed that exposure of pregnant mice to escalating dose of pritelivir did not result in bladder epithelial hyperplasia, likely due to lower susceptibility of mice to the effect of sulfonamide compounds on this tissue than rats or shorter exposure to pritelivir than in the chronic toxicity study on rats. On the other hand, regeneration of the affected urinary bladder epithelium is also possible between GD 15 (the day of the last dose) and GD 18 (the day of animal sacrifice). Regardless, the lack of rodent-specific adverse effects of pritelivir supports the use of mice in the pEFD studies of this novel drug. No pathological changes were noted in the placental tissue. No pritelivir-related fetal external and visceral evaluations were noted. Both maternal and fetal NOAEL of pritelivir in CD1-IGS mice in our pEFD study was 200 mg/kg/day, which was the highest dose tested and resulted in a 20-fold higher plasma exposure of maternal animals than in humans at a clinical dose of 100 mg/day.

The PK profile of pritelivir in plasma of non-pregnant mice treated with pritelivir at 10 mg/kg/day dose at steady state resembled that of a single dose reported previously^26^ which was anticipated from the relatively short reported half-life of 7 h of the drug in mice in comparison to the 24 h dose interval^26^. The T_max_ at steady state at this dose was also consistent with the value reported for a single dose^26^. It appears that pregnancy did not affect the PK parameters of pritelivir in mice at steady state (Table S6). However, our data revealed a split-peak PK profile of pritelivir in the plasma of pregnant mice treated with 200 mg/kg/day (Fig. 3), with the second peak representing the C_max_, while data from nonpregnant female mice yielded monophasic PK profile at this dose (Figure S1). These results suggest pregnancy-associated changes in the absorption and elimination rates of pritelivir with the dose escalation to 200 mg/kg/day, and a higher second peak likely indicates delayed gastric emptying of the drug in pregnancy in mice due in part to elevated progesterone levels^27,28^.

One of the endpoints of our toxicokinetic study in CD1-ISG mice was determination of pritelivir distribution in the major organs. Data revealed similar exposure levels of major maternal organs to the drug in pregnant vs. nonpregnant female mice (Tables S7&S8) judged by the AUC_ss_ as well as C_max_ values. The tissue AUC_ss_/plasma AUC_ss_ ratios were the lowest for the brain as compared to other organs ranging from 0.23 to 0.3 and were relatively constant irrespective of pritelivir dose. These ratio values were within the range of 0.2–0.5 which is considered “medium” and is indicative of moderate penetration of the drug across the blood-brain barrier^29^. A recent study classified permeability of pritelivir in the brain of mice as poor based on the brain/plasma concentration ratio of 0.05 (which is below the threshold of 0.1) observed in mice at 4 h post-dose following oral administration of a single 10 mg/kg dose of pritelivir^29^. Higher brain/plasma ratio for pritelivir observed in our study could be explained by repeat dose administration aiming to achieve steady state levels of pritelivir. The presence of pritelivir in the maternal brain was also confirmed by DESI-MSI which showed that the drug is distributed primarily in brain ventricles (Figure S2A&B).

As part of our toxicokinetic study in pregnant mice, we also investigated the disposition of pritelivir into the placenta and the fetus. While LC-MS/MS analysis of tissue homogenates showed that the drug crosses the placenta and appears in the fetal tissues, data suggest limited passage of pritelivir across the placenta in mice (Table 3). DESI-MSI revealed that within the fetus pritelivir is distributed primarily in the liver (Fig. 4 and S2). DESI-MSI technique corroborated our LC-MS/MS findings revealing higher signal intensity of pritelivir in the placental tissue as compared to the fetus. De Vries et al.^30^ reported that pritelivir is a substrate of P-glycoprotein (P-gp) and Breast Cancer Resistance protein (BCRP) efflux transporters^30^ which are localized in the apical (maternal facing) membrane of murine as well as human placentas^31^. Moreover, our recent study on the passage of pritelivir across the term human placenta ex vivo showed that the transfer of this drug in the fetal-to-maternal direction was greater than in the maternal-to-fetal direction, suggesting a protective role of placental transporters in limiting fetal exposure to the drug^32^. Thus, P-gp and BCRP and possibly other efflux transporters in the murine placenta could have played a role in the distribution of pritelivir between maternal plasma and placental tissue in the current study, along with other contributing factors^31^. A prior report regarding C^14^-labeled amenamevir (a helicase-primase inhibitor approved in Japan to treat varicella zoster and HSV) in mice at GD 12^33^ suggest the possibility of greater accumulation of amenamevir and its metabolites in the placenta than we had observed for pritelivir in our toxicokinetic study. However, it is important to note that this is not a direct comparison due to lack of metabolite data in our results as the quantification of pritelivir metabolites^19^ in the samples from pregnant mice and fetoplacental compartment was outside of the scope of our toxicokinetic study. Another study reported concentrations of acyclovir in maternal plasma, umbilical vein plasma as well as amniotic fluid samples collected at delivery from women receiving 500 mg oral valacyclovir twice daily or 400 mg acyclovir three times a day^34^. The data indicated that acyclovir crosses the human placenta in vivo close to term which was based on the acyclovir concentrations in the umbilical vein plasma and amniotic fluid in relation to acyclovir quantified in all corresponding maternal plasma samples^34^.

In conclusion, once-daily dosing of up to 200 mg/kg of pritelivir via oral gavage during the period of organogenesis did not cause maternal toxicity and did not influence the early embryonic development of mice. Moreover, lower concentrations of pritelivir in the placenta and fetus of mice than in the maternal plasma showed that the drug does not accumulate in the fetoplacental compartment and suggests less-than-extensive disposition of pritelivir across the placental barrier. Taken together, our findings support further preclinical studies of this novel anti-herpes virus drug pritelivir for its use in pregnancy.

## Methods

### Ethical considerations

The animal studies in the current report were designed following the guidelines of the International Conference on Harmonization (ICH) M3(R2)^14^, ICH-S5(R3) (Detection of Toxicity to Reproduction for Human Pharmaceuticals)^15^ and recommendations of Wise et al.^16^.

The pEFD study was conducted at Labcorp Early Development Laboratories, Inc., which is accredited by the Association of Assessment and Accreditation of Laboratory Animal Care (AAALAC). All procedures in the study protocol were following the Animal Welfare Act, the Guide for the Care and Use of Laboratory Animals, and the Office of Laboratory Animal Welfare and were approved by the local Institutional Animal Care and Use Committee (IACUC) (Protocol Number 04512-B_2023). While the pEFD study was a non-regulatory study, and as such, did not require compliance with the principles of US Food and Drug Administration (FDA) Good Laboratory Practices (GLP), the study was conducted in accordance with the standard of operating procedure (SOP) fully comparable with international GLP standards^16^ meeting the requirements stated by the M3(R2) guidance, Note 4^14^.

The satellite toxicokinetic study was conducted at the University of Texas Medical Branch (UTMB) at Galveston and was approved by the IACUC at UTMB (Protocol Number 2208049) and was in accordance with the guidelines published by the US National Institutes of Health Guide for the Care and Use of Laboratory Animals (National Research Council (US) Committee for the Update of the Guide for the Care and Use of Laboratory Animals.^35^.

### Chemicals and dosing

Pritelivir (BAY 57-1293, CAS No. 348086-71-5, Batch No. 0676813, purity ≥ 98.8%) and pritelivir-d_4_ (BAY 57-1293-d_4_) were purchased from Cayman Chemical Co. (Ann Arbor, Michigan). Carboxymethylcellulose (CMC) sodium salt (low viscosity, batch no. SLCP0901) was purchased from Sigma-Aldrich (St Louis, MO).

Low and medium dose levels of 10 and 60 mg/kg/day for pritelivir were selected based on the available data on the PK profile of this drug in nonpregnant mice after single oral applications of 10 mg/kg and 60 mg/kg. Since dose-dependent hyperplasia of the urinary bladder epithelium was reported in rats in the exploratory 4-week chronic toxicity study of 30, 100 and 300 mg/kg/day, and was hypothesized to be rodent-specific^23^(as mentioned above), we decided to establish a high dose level of 200 mg/kg/day to follow recommended spacing between the doses in drug-treated groups^16^ and avoid possible excessive maternal toxicity that could interfere with the interpretation of any embryo-fetal findings.

Previous preclinical studies in rodents and monkeys utilized CMC as a vehicle for preparation of pritelivir free base formulations for oral gavage^23,36^. In our investigation, the suspensions of pritelivir free base were freshly prepared each day in a 1% aqueous CMC solution in purified water (w/v) to achieve 1 mg/mL, 6 mg/mL and 20 mg/mL final concentrations for oral gavage in mice. The formulations were stored in the refrigerator set to maintain 2 to 8 °C and protected from light while continuously stirring until removed for dosing. Prior to initiation of the pEFD study, we confirmed the stability of the drug in aqueous solution for at least 24 h at ambient temperature as well as at 4 °C protected from light (see *Supplementary Information* for details). Vehicle control was prepared and apportioned for daily use.

### Animals, study designs and parameters evaluated

#### pEFD study

For the pEFD study, a total of forty CD1-IGS time-mated female mice were received from Charles River Laboratories (Raleigh, North Carolina). The animals were shipped prior toGD 4 and allowed to acclimate for 3 or 4 days upon arrival.

The mice were individually housed in polycarbonate cages with Diamond Soft bedding with unlimited access to food (Certified Rodent Diet #2016 C, Envigo RMS, Inc.) and water. Environmental controls were set to maintain a temperature range of 20 to 26 °C, a relative humidity range of 30 to 70%, 10 or greater air changes/hour, and a 12-hour light/12-hour dark cycle. Any variations to these conditions are maintained in the raw data and had no effect on the outcome of the study. Environmental enrichment was provided as a variety of cage-enrichment devices and dietary enrichment. Animals were randomly allocated to treatment groups receiving 0 (i.e., vehicle alone), 10, 60 or 200 mg/kg/day pritelivir daily from GD 6 through 15 via oral gavage and the dose was adjusted based on the most recent recorded body weight. The pritelivir suspension concentrations were prepared as described above so the volume for oral gavage in mice did not exceed 5 mL/kg. Animals were 11 weeks old at the initiation of dosing, and their body weights ranged from 27.0 to 35.7 g and were similar among the study groups at randomization (see statistical analysis section).

The animals were checked twice daily (a.m. and p.m.) for mortality, abnormalities, and signs of pain or distress. Cage side observations were also performed during dosing intervals at approximately 6 h post-dose for each subject, as well as detailed observations made on GD 4, 6, 12, and 18.

Animals were weighed on GD 4, 6, 9, 12, 15, and on GD 18 (terminal body weight). Food consumption (quantitative) was measured for each animal on GD 4, 6, 9, 12, 15, and 18. Food consumption intervals documented as spilled food were recorded but excluded from summary values for affected animals.

At GD 18, animals were sacrificed via carbon dioxide inhalation, followed by exsanguination. The placenta and amniotic sac were examined for abnormalities, and each placenta was excised and weighed. Placentas were collected and individually identified for approximately 50% of live fetuses selected for Wilson’s sectioning. When no fetuses were observed *in utero* and implantation sites were not apparent when pressing the uteri between glass plates, the uterus was placed in ammonium sulfide solution^37^. The uterus from each pregnant animal was excised, weighed and examined for the number and placement of live and dead fetuses, the number of early or late resorptions, and any abnormalities. One animal from the vehicle control group delivered prior to sacrifice on GD 18 and was necropsied, to the extent possible, as indicated previously for terminally sacrificed animals. The right and left ovaries from each pregnant female were examined for the number of corpora lutea. Each animal was examined macroscopically for abnormalities of the external features of the carcass; external body orifices; abdominal, thoracic, cranial, pelvic, and oral cavities; organs; and tissues. The following tissues were weighed from each dam: liver (including gall bladder, drained), uterus (gravid with cervix at time of cesarean section), urinary bladder and placentas. The histopathology slides were prepared from uterus, cervix, urinary bladder and placentas from 50% of live fetuses selected for Wilson’s sectioning (see below). The specimens were preserved with 10% neutral-buffered formalin, embedded in paraffin (each placenta was imbedded in a separate block), sectioned at 5 μm (for placenta, cross-section was performed though the central portion and trimmed; for urinary bladder, longitudinal sections from both halves were included) and stained with hematoxylin and eosin (H&E).

For fetal examinations, each live fetus was weighed, the sex was recorded, and each fetus was examined for external abnormalities. Live fetuses were euthanized via injection with barbiturate, followed by exsanguination and processed for visceral examination, in which internal organs of the thoracic and abdominal cavities were examined in the fresh state using modified Staples’ technique^38^. For half of the fetuses (50% from each litter as selected by a computer) heads were removed, stored frozen on dry ice, and cross-sectioned using Wilson’s sectioning technique^39^.

Findings were classified as follows: (a) malformations, defined as developmental deviations which are macroscopic structural changes that could potentially affect survival, development or function, and (b) variations, defined as normal anatomical differences within the population and are thought to have no effect on body conformity or the wellbeing of an animal^40^.

#### Toxicokinetic study

For the satellite toxicokinetic study, we utilized the total of 75 CD1-IGS time-mated female mice and 38 CD1-IGS nonpregnant female mice. Animal sources (as well as GD for the time-mated female mice) at shipping were the same as described above for the pEFD study, and environmental conditions, diet, water and environmental enrichment were similar to those described above.

#### Toxicokinetic study on time-mated female mice

At the initiation of dosing, time-mated animals were 11–12 weeks old, and body weights ranged from 18 to 41.5 g. The mice were randomly allocated to the treatment groups receiving 10 mg/kg/day and 200 mg/kg/day pritelivir daily from GD 9 through GD 13 via oral gavage; the duration of treatment was selected to assure the administered drug reaching steady state at the time of a scheduled sacrifice and was based on the reported half-life of 7 h for pritelivir administered to nonpregnant mice in a single dose of 10 mg/kg^26^. Three experiments were conducted for each dose and utilized *n* = 6–14 animals covering *n* = 5–6 time points, as described below. The administered dose was adjusted for body weight, which was assessed in animals at arrival on GD 4 and subsequently at GD 9, 11 and 13. One time-mated mouse was allocated to the vehicle control group. Clinical observations were made approximately 4 h after dosing (the anticipated time of T_max_)^26^. The following animal conditions were recorded: mortality, moribundity, and clinical signs of toxicity were scored as described previously^37^. Food consumption was not measured as it was within the scope of the pEFD study.

At GD 13, mice were humanely sacrificed via carbon dioxide inhalation, followed by bilateral thoracotomy, and blood samples were collected from a cardiac puncture at 1, 3, 4, 8, and 24 h post-dose for the drug-treated mice and at 4 h post-vehicle administration for the control animal. Blood was centrifuged, and plasma stored at -80 °C until analysis using LC-MS/MS (see below). Uteri were opened to confirm pregnancy status; fetuses were removed and euthanized via decapitation. Fetuses and placentas were weighed, then *n* = 4 placentas and fetuses from each litter were used for the analysis of pritelivir. Brain, heart, liver, spleen, lungs and kidneys were collected from each maternal animal, weighed and stored at -80 °C in the vacuum-sealed bags until analysis using LC-MS/MS (see below). We allocated one pregnant maternal animal that was sacrificed at 4 h postdose in the 10 mg/kg/day treatment group and 8 h postdose in the 200 mg/kg/day group (i.e., respective anticipated plasma T_max_ for each dose) for the collection of tissue samples to be analyzed using DESI-MSI technique. Accordingly, brains and livers of maternal animals and *n* = 3 fetuses with corresponding placentas were frozen on dry ice and processed further as described below. The rest of maternal organs from these two mice and fetuses and placentas (*n* = 4 each) as well as *n* = 4 samples of amniotic fluid from individual amniotic sacs were collected as described above for the LC-MS/MS analysis. Two fetuses and placentas from the vehicle-treated pregnant mouse were also reserved for DESI-MSI analysis and processed accordingly.

#### Toxicokinetic study on nonpregnant female mice

At the initiation of dosing, the animals were 10–12 weeks old, and body weights ranged from 23 to 31 g. The mice were randomly allocated to the treatment groups receiving 10 mg/kg/day and 200 mg/kg/day pritelivir for five days. Two experiments were conducted for 10 mg/kg/day dose and one for 200 mg/kg/day dose, each experiment utilized *n* = 5–15 animals covering *n* = 5–6 time points, as described above for the time-mated mice. The administered dose was adjusted for body weight, which was assessed in animals at arrival and subsequently at the time of the first dose, and day three and five of dosing. Clinical observations were made in the same manner as described above for time-mated mice. Mice were humanely sacrificed on day five of dosing and blood samples as well as tissue samples from maternal animals were collected and stored until LC-MS/MS analysis as described above for time-mated animals.

#### Determination of pritelivir concentration in mice samples by LC-MS/MS

The quantitative determination of pritelivir in plasma, amniotic fluid and tissue samples was conducted using LC-MS/MS method that we developed and validated; the equipment and analytical conditions were described in detail in our previous report^32^. The protocol for sample processing as well as preparation of standards and quality control samples is outlined in *Supplementary Information*.

### DESI-MSI

#### Tissue samples preparation

Frozen embryos and placentas from mice were placed into a cryomold and embedded in 7% (w/v) gelatin (Fisher Scientific, Waltham, MA) dissolved in Optima LC/MS-grade water (Fisher Scientific). The gelatin solution was first prepared at 50 °C and after cooling to 25 °C, it was added to the cryomold until the tissues were completely submerged. The gelatin block was rapidly frozen on dry ice in a sealed styrofoam container and stored at − 80 °C until sectioning. Murine brain and liver tissues required no prior embedding and were sectioned directly.

Twelve-micrometer-thick tissue sections were prepared using a Leica CM1850 cryostat (Leica Microsystems, Illinois, USA) and mounted onto Superfrost Plus microscope glass slides (Fisher Scientific). Tissue sections were desiccated under vacuum for 10 min using a vacuum desiccator, then individually sealed in zipper-lock bags and stored at − 80 °C until further use.

#### DESI MSI analysis and data acquisition

DESI–multiple reaction monitoring (MRM) analyses were performed using a Xevo TQ Absolute mass spectrometer (Waters Corporation, Milford, MA, USA) equipped with a DESI XS ion source (Waters Corporation). A 98:2 (v/v) methanol/water spray solvent was delivered at 2 µL/min via a µBSM nano pump (Waters Corporation). Key source parameters were as follows: source temperature, 150 °C; nebulizer gas pressure, 13 psi; capillary voltage, 0.7 kV; cone voltage, 20 V; pixel size, 35 μm; and acquisition rate, 10 scans/s (dwell time, 12 ms per MRM transition). Pritelivir was detected using its [M + H]⁺ precursor-to-product ion transition at m/z 403.1 → 196.1, To correct for spatially dependent ionization variability, ion signals were normalized on a pixel-by-pixel basis to the stable isotope-labeled internal standard pritelivir-d_4_ ([M + H]⁺, m/z 407.1 → 200.1), added to the spray solvent at 1 µg/mL. A cone voltage of 20 V and collision energy of 25 eV were applied uniformly to both analytes. Sprayer nozzle geometry was systematically optimized to maximize pritelivir signal intensity while preserving spatial resolution. The optimized configuration comprised a spray angle of 80 °C, a sprayer-to-surface distance of 1 mm, a sprayer-to-inlet distance of 5 mm, and an MS inlet height of 0.3 mm above the tissue section.

Data acquisition was performed using MassLynx v4.2 and the DESI Method Editor (Waters Corporation). Imaging data were processed and visualized using High Definition Imaging (HDI) software v1.8 (Waters Corporation). Data files were exported from HDI in imzML format for downstream analysis. Further visualization and region-of-interest (ROI) ion signal quantitation were carried out in SCiLS pro 2026bLab software (Bruker, Bremen, Germany), using adjacent H&E-stained sections as optical references to delineate anatomical ROIs. H&E-stained sections were prepared by the UTMB Research Histology Core Facility following standardized protocols.

### Data and statistical analysis

#### pEFD study

Data were calculated as mean ± S.D.; and statistical analysis was performed using SAS software, version 9.4 (M8) of SAS System for Windows Copyright© [2023] SAS Institute Inc. Statistical significance was considered attained at *P* < 0.05. Data from animals that were found not pregnant at the end of the study were excluded from the statistical analysis.

There was no difference among the mean body weight for each group of animals upon randomization (namely, vehicle control and three drug-treated groups) prior to dosing as determined by the F-test, and Levene’s test confirmed homogeneity of variances across the groups. At the end of the study, maternal body weight as well as weight change and food consumption were compared using analysis of variance (ANOVA) and, if significance was attained, Dunnett’s post-hoc test was conducted to compare drug-dosed and control groups; *t*-test pairwise comparison was conducted for drug-dosed groups. Prior to ANOVA, data were first analyzed with Levene’s test for homogeneity, and if Levene’s test attained significance, Kruskal-Wallis nonparametric ANOVA was used for multiple comparisons and Wilcoxon Rank Sum Test for two-group comparisons.

The proportions of litters with fetal observations were analyzed using Fisher’s Exact test (upper tail). The percentage of fetuses with fetal observations was analyzed using a Kruskal-Wallis nonparametric ANOVA and, if significance was attained, pairwise comparisons of each drug-treated group with the control group were made using the Wilcoxon Rank Sum Test.

Data on gravid uterine weight and corrected body weight were tested for homogeneity of variances using Levene’s test. If homogeneity of variances was assumed, groups were compared using one-way ANOVA followed by Dunnett’s post-hoc test, if applicable. In case Levene’s test attained significance, groups were compared with Kruskal-Wallis nonparametric ANOVA. Analysis of fetal weights (male and female separately and combined) was performed using analysis of covariance (ANCOVA), followed by Dunnett’s post-hoc test, if applicable. Mean numbers of corpora lutea, implantation sites, early and late resorptions, dead fetuses, live fetuses, percent pre- and post-implantation loss, and percent male fetuses were compared using Kruskal-Wallis nonparametric ANOVA followed by pairwise comparisons of each pritelivir-treated group with the control group using the Wilcoxon Rank Sum Test, if applicable.

#### Toxicokinetic study

Data were calculated as mean ± S.D.; statistical analysis was performed using SPSS, version 28 for Windows (SPSS Inc, Chicago, IL). The results were considered statistically significant when *P* < 0.05. Data from animals that were found not pregnant at the end of the in-life portion of the toxicokinetic study on time-mated mice were excluded from the analysis of the data set obtained from pregnant animals and were added to the data set obtained from the group of non-pregnant mice.

There was no difference among the mean body weight of non-pregnant animals allocated to each toxicokinetic experiment as determined by the F-test, and Levene’s test showed homogeneity of variances across the groups. The PK data were analyzed with Kinetica software (v. 5.1, Thermo Fisher Scientific, Waltham, MA)^41,42^ and included C_max_ and T_max_ as determined by visual examination of the pritelivir concentration vs. time curve for each experiment utilizing multiple animals (*n* = 1–3 mice per time point, total *n* = 3 experiments for each dose in pregnant and nonpregnant mice). For each experiment, the plasma and tissue samples were collected between 1 h and 24 h after the last of five daily doses, so steady state was assumed. Accordingly, in the calculations of the AUC_ss_, the concentration at the time of the last dose on day five (i.e., time 0) was assumed to match the concentration measured 24 h post dose. The AUC_ss_ values were determined by non-compartmental analysis utilizing the trapezoidal method. For maternal organs, the tissue AUC_ss_/plasma AUC_ss_ ratios were derived to assess the disposition of the drug. Student *t*-test was utilized to compare the corresponding PK parameters obtained from pregnant and nonpregnant female mice for each dose.

Pritelivir was also quantified in *n* = 4 individual placentas and fetuses from each litter of pregnant mice. The average values from each litter were subsequently used to build a tissue concentration-time curve, and data from multiple dams/litters were used to derive AUC_ss_ in the placental and fetal tissues. Amniotic fluid samples from individual amniotic sacs samples (*n* = 4) were available from one animal for each of 10 mg/kg/day and 200 mg/kg/day dose groups and collected at the time of anticipated plasma T_max_ of pritelivir for each dose (4 h and 8 h, respectively). The disposition of pritelivir in the fetoplacental compartment was assessed as follows: (a) The placental AUC_ss_/plasma AUC_ss_ and fetal AUC_ss_/plasma AUC_ss_ ratios for each experiment; (b) the tissue/plasma ratios calculated for the corresponding concentrations at T_max_ for plasma as well as at T_max_ for placenta and the fetus for each experiment; and (c) the amniotic fluid/plasma concentration ratio calculated using the values obtained from the corresponding maternal animals.

## Supplementary Information

Below is the link to the electronic supplementary material.

Supplementary Material 1

## Data Availability

The data that support the findings of this investigation are available on request from the corresponding author, V.B.

## Abbreviations

AAALAC: Association of Assessment and Accreditation of Laboratory Animal Care
AUC_ss_: Area under the concentration-time curve at steady state
BCRP: Breast cancer resistance protein
C_max_: Maximum plasma concentration
DESI -MSI: Desorption electrospray ionization mass spectrometry imaging
DMSO: Dimethyl sulfoxide
GD: Gestational day
HED: Human equivalent dose
HSV: Herpes simplex virus
HUVEC: Human umbilical vein endothelial cells
IACUC: Institutional Animal Care and Use Committee
LC-MS/MS: Liquid chromatography – mass spectrometry
NOAEL: No-observed-adverse-effect-level
GLP: Good laboratory practice
pEFD: Preliminary embryo-fetal development
P-gp: P-glycoprotein; PK Pharmacokinetics
SOP: Standard of operating procedures
T_max_: Time of the maximum plasma concentration
