# Supplementary material for: Preliminary embryo-fetal developmental toxicity study of pritelivir in CD1-IGS mice
Source: Sci Rep. 2026 Jun 21;16:37725. doi: 10.1038/s41598-026-57669-x (PMC13754608; doi:10.1038/s41598-026-57669-x)
Supplement: Supplementary file 1 [file 41598_2026_57669_MOESM1_ESM.pdf]

Preliminary embryo-fetal developmental toxicity study of pritelivir in CD1-IGS mice

Tatiana N. NANOVSKAYA, PhD[a], Svetlana L PATRIKEEVA, MS[a], Xiaoming WANG, PhD[a], Erik RYTTING, PhD[a], Bandana BERA, MS[b], Sydni C ZACHARIAS, MS[b], Brendan PRIDEAUX, PhD[b], and Valentina F BRYANT, PhD[a]

[a]Maternal-Fetal Pharmacology and Bio-Development Laboratories, Department of Obstetrics & Gynecology, The University of Texas Medical Branch,
301 University Boulevard, Galveston, TX 77555-0587, USA.

[b]Department of Neurobiology, The University of Texas Medical Branch,
301 University Boulevard, Galveston, TX 77555-0620, USA.

SUPPLEMENTARY MATERIALS

Preliminary embryo-fetal developmental toxicity study: assessment of stability of pritelivir in aqueous solution

Preparation of stock solutions and calibration working solution

Pritelivir was dissolved in dimethyl sulfoxide (DMSO) to obtain 1 mg/mL stock solution, which was subsequently diluted to 100 µg/mL and 5 µg/mL solutions with water. The resulting stock solutions were aliquoted in 2 mL glass vials. The aliquoted stock solutions were stored in the refrigerator (4°C) or bench top (ambient temperature, 22-25°C) protected from natural light.

The calibration working solution of pritelivir were prepared by diluting the stock solution to 2.5, 12.5, 125, 625, 1250, 3750, and 5000 ng/mL. Pritelivir-d_4, which served as the internal standard for the quantitative determination of pritelivir, was prepared by dissolving the standard in 30% (v/v) methanol (final concentration was 56.4 ng/mL).

Sample preparation and data analysis

The 100 µg/mL and 5 µg/mL stock solutions of pritelivir were diluted 40 folds with water resulting in working solutions. For the analysis by LC-MS/MS, the following samples were prepared in triplicates for each of the concentrations of pritelivir: 10 µL of the working solution of

the drug were mixed with 10 µL of the internal standard stock solution and 100 µL of the initial mobile phase. The resulting samples were injected into the LC-MS/MS system. The injection volume was 25 µL.

The calibration standards were prepared in a similar manner for each analysis (that is, analysis at time 0 and time 24 h) to generate calibration curves for pritelivir quantification in the tested samples. The concentrations of pritelivir determined in the 24 h samples were expressed as percentage of those determined at time 0 to assess the stability of the drug under the tested conditions.

Toxicokinetic study: preparation of plasma, amniotic fluid and tissue samples for quantitative determination of pritelivir

Sample preparation

Plasma samples were processed as follows: a defined volume of a sample was first diluted with phosphate buffered saline (PBS) (v/v) either 20-fold (for the samples obtained from dams that received the 10 mg/kg/day pritelivir dose) or 100-fold (for the samples obtained from animals that received the 200 mg/kg/day dose). Amniotic fluid samples were diluted in a similar manner 5- and 50-fold, respectively. Then 20 µL of each diluted sample was placed in a tube, spiked with 10 µL of working solution of the IS and mixed for 5 s. The proteins were precipitated by adding 100 µL of methanol to the tube and vigorously shaking for 5 min. Then the sample was centrifuged for 20 min at 15,000 g, the supernatant was transferred to an high performance liquid chromatography (HPLC) vial, and 2 µL were injected for analysis.

Placentas and fetuses (n=4 of each tissue from each dam that received 10 mg/kg/day pritelivir) were processed individually by homogenizing in PBS with a ratio 1:4 (w/v) (placenta) and 1:3 (w/v) (fetus) for 20-40 s using a Tissue-Tearor 985370-07 Homogenizer at a speed 4-8. Then 20 µL of homogenate were processed as described above for plasma. For the placentas and fetuses obtained from animals that received 200 mg/kg/day, the placentas and fetuses were homogenized in PBS with a ratio of 1:4 (w/v), and the homogenates were subsequently diluted

with PBS to achieve final dilution with the ratio of 1:24 (v/v) before processing as described above for plasma.

Maternal organs were homogenized in a similar manner in PBS with a ratio of 1:4 (w/v), and the homogenates subsequently diluted with PBS to achieve final dilutions with the ratios of 1:24 and 1:99 (w/v) for the 10 mg/kg/day and 200 mg/kg/day treatment groups, respectively; then 20 μL of homogenate were processed as described above for the LC-MS/MS analysis.

Preparation of standards and calibration samples

Calibration and quality control samples were prepared using respective tissues homogenates from the control animal of our study, and murine plasma (Innovative Grade US Origin Mouse CD1 Plasma, Innovative Research, Inc.). The calibration curve was prepared with the final concentrations of pritelivir ranging from 0.04 μg/mL to 2.5 μg/mL. Each analysis of the samples was accompanied by quality control samples at high, medium and low concentration levels.

LC-MS/MS conditions

The equipment and analytical conditions were described in detail in our recent report[12].

Table S1. Summary of body weight of pregnant CD1-IGS mice.

	Pritelivir dose, mg/kg/day				
	0 (control)	10	60	200	*P*-value
Gestational day					
4	30.6±1.78	30.2±1.83	29.4±1.54	30.9±0.95	0.299
6	31.5±1.75	31.1±1.47	30.8±1.57	32.4±1.01	0.791
9	33.0±2.46	33.0±1.24	32.2±1.71	33.2±1.77	0.740
12	37.2±4.36	38.8±1.97	37.9±2.83	37.9±2.42	0.778
15	44.1±7.87	48.3±3.59	45.2± 4.40	45.8±4.01	0.451
18	52.3±14.12	62.1± 6.50	56.1±6.17	57.1±5.52	0.216*

Data presented as mean±S.D. of n=9, 8, 6 and 9 maternal animals per treatment group (0, 10, 60, and 200 mg/kg/day dose, respectively, administered from gestational day 6 through 15). Data were first analyzed using Lavene's test for homogeneity, subsequently the groups were compared using either one-way ANOVA (equally variance assumed) or Kruskal-Wallis test (equal variances not assumed, the reported *P*-value marked with *). The resulting *P*-values were greater than 0.05, therefore the respective post-hoc analysis is not reported.

Table S2. Summary of body weight change of pregnant CD1-IGS mice.

	Pritelivir dose, mg/kg/day				
	0 (control)	10	60	200	*P*-value
Phase day (from X to X)					
4 to 6	0.89±0.76	0.91±0.81	1.43±0.71	0.51±0.84	0.200
6 to 9	1.49±1.82	1.89±0.57	1.37±0.46	1.82±1.16	0.804
9 to 12	4.21±2.05	5.76±1.06	5.70±1.77	4.67±1.16	0.145
12 to 15	6.87±3.59	9.53±2.19	7.38±2.34	7.94±1.71	0.210
15 to 18	8.17±8.38	13.79±3.51	10.82± 2.24	11.30±1.98	0.258*
6 to 15	12.6±6.98	17.18± 3.15	14.45±3.77	14.43±3.43	0.277
6 to 18	20.8±12.9	30.96±6.26	25.27±5.81	25.73±4.94	0.193*

Data presented as mean±S.D. of n=9, 8, 6 and 9 maternal animals per treatment group (0, 10, 60, and 200 mg/kg/day dose, respectively, from gestational day 6 through 15). Data were first analyzed using Lavene's test for homogeneity, subsequently the groups were compared using either one-way ANOVA (equally variance assumed) or Kruskal-Wallis test (equal variances not assumed, the reported *P*-values marked with *). The resulting *P*-values were greater than 0.05, therefore the respective post-hoc analysis is not reported.

Table S3. Maternal outcomes

**Pritelivir dose (mg/kg/day)**	**0 (control)**	**10**	**60**	**200**
Number of pregnant animals	9	8	6	8
Number of death or sacrificed moribund	0	0	0	0
Number of aborted or with no viable fetuses	1	0	0	0
Clinical Observations	-	-	-	-
Macroscopic Observations	-	-	-	-
Organ (Liver/Gall Bladder) Weights	-	-	-	-
Microscopic Observations	-	-	-	-

- , No noteworthy findings; GD, gestation day

Table S4. Summary of cesarean section data and mean fetal data

**Pritelivir dose, mg/kg/day**		**0 (control)**	**10**	**60**	**200**
**Summary of cesarean section data**					
Number of females pregnant at cesarean section	(n)	9	8	6	9
Corpora lutea	(n)	9	8	6	9
	Mean±S.D.	12.6±5.34	15.3±3.49	13.2±1.17	13.3±3.39
Implantation sites	(n)	9	8	6	9
	Mean±S.D.	11.6±5.13	13.6±2.20	12.8±1.17	13.0±3.08
Preimplantation loss	(n)	9	8	6	9
	Mean±S.D.	1.0±1.41	1.6±2.77	0.3±0.52	0.3±0.71
Preimplantation loss (%)	(n)	9	8	6	9
	Mean±S.D.	7.59±13.077	8.54±14.140	2.47±3.834	2.00±4.204
Early resorptions	(n)	9	8	6	9
	Mean±S.D.	0.9±1.05	0.4±0.52	1.0±2.45	0.6±0.88
Late resorptions	(n)	9	8	6	9
	Mean±S.D.	0.4±0.73	0.1±0.35	0.0±0.00	0.0±0.00
Total resorptions	(n)	9	8	6	9
	Mean ± S.D.	1.3±1.22	0.5±0.76	1.0±2.45	0.6±0.88
Resorption rate per animal %	(n)	9	8	6	9

	Mean±S.D.	18.8±31.50	4.1±7.05	8.3±20.41	3.8±5.92
Dead fetuses	(n)	9	8	6	9
	Mean±S.D.	0.2±0.44	0.0±0.00	0.0±0.00	0.0±0.00
Postimplantation loss	(n)	9	8	6	9
	Mean±S.D.	1.6±1.42	0.5±0.76	1.0±2.45	0.6±0.88
Postimplantation loss (%)	(n)	9	8	6	9
	Mean±S.D.	20.69±31.405	4.11±7.052	8.33±20.412	3.72±5.921
Live fetuses	(n)	9	8	6	9
	Mean±S.D	10.0±4.97	13.1±2.53	11.8±3.06	12.4±2.79
Gravid uterine weight	(n)	8	8	6	9
	Mean±S.D	17.92±8.739	24.63±3.279	21.17±4.911	20.53±3.753
Corrected body weight (carcass weight)	(n)	8	8	6	9
	Mean±S.D.	36.33±5.509	38.39±2.360	34.28±2.230	35.92±2.063
Corrected weight change	(n)	8	8	6	9
	Mean±S.D.	5.47±5.370	8.09±2.952	4.93±1.622	5.04±1.843
**Summary of mean fetal data**					
% Male fetuses	(n)	9	8	6	9
	Mean±S.D.	42.0±24.58	54.6±12.05	54.3±11.91	48.8±14.24
Mean fetal weight	(n)	8	8	6	9

	Mean±S.D.	1.461±0.1373	1.386±0.1075	1.353±0.0411	1.306±0.1985
	Adj. Mean S.D.	1.433±0.1373	1.414±0.1075	1.342±0.0411	1.314±0.1985
Mean weight - male fetuses	(n)	8	8	6	9
	Mean±S.D.	1.498±0.1791	1.410±0.0916	1.361±0.0327	1.313±0.1830
	Adj. Mean S.D.	1.471±0.1791	1.436±0.0916	1.351±0.0327	1.320±0.1830
Mean weight – female fetuses	(n)	8	8	6	9
	Mean±S.D.	1.427±0.1213	1.358±0.1401	1.345±0.0541	1.295±0.2099
	Adj. Mean±S.D.	1.398±0.1213	1.387±0.1401	1.334±0.0541	1.302±0.2099

Data presented as mean±S.D. and adjusted mean±S.D. where applicable.

The following formulas were used to calculate select parameters:

$$\text{Mean \% Preimplantation Loss} = \frac{\text{(corpora lutea/litter - implantation sites/litter)}}{\text{corpora lutea/litter}} \times 100\%$$

$$\text{Mean \% Postimplantation Loss} = \frac{\text{(dead fetuses/litter + resorptions/litter)}}{\text{implantation sites/litter}} \times 100\%$$

$$\text{Sex Ratios} = \frac{\text{(fetuses [male:female] in each group)}}{\text{total fetuses/group}} \times 100\%$$

Table S5. LabCorp Early Development Laboratories Inc. historical control data (2014-2019) on embryo-fetal development of CD1-IGS mice. Summary incidence of visceral anomalies.

No. of studies: 6

No. of litters: 113

No. of fetuses: 707

		Proportion of litter with observation (%)			Mean incidence (% per litter)		
		Mean±S.D.	Min	Max	Mean±S.D.	Min	Max
**Abdomen**	Abdominal cavity–fluid filled	0.83±2.04	0	5	0.11±0.27	0	0.65
**Blood vessel**	Umbilical artery–left-sided	18.17±8.59	5	29	3.30±2.09	0.65	5.92
**Kidney**	Kidney–dilated	0.83±2.04	0	5	0.13±0.31	0	0.76
	Kidney–discolored	0.83±2.04	0	5	0.10±0.23	0	0.57
	Kidney –misshapen	0.83±2.04	0	5	0.10±0.23	0	0.57
**Lung**	Intermediate lobe–absent	1.50±3.67	0	9	0.25±0.62	0	1.52
**Spleen**	Spleen–supernumerary	0.67±1.63	0	4	0.10±0.23	0	0.57
**Stomach**	Distended	2.17±5.31	0	13	0.30±0.73	0	1.79
**Thoracic cavity**	Thoracic cavity–fluid filled	0.83±2.04	0	5	0.11±0.27	0	0.65
**Ovary**	Ovary–cyst	1.0±2.46	0	6	0.26±0.64	0	1.56

Table S6. Pharmacokinetic parameters of pritelivir in the maternal plasma of pregnant and nonpregnant female CD1-IGS mice after oral administration of multiple doses of 10 mg/kg/day and 200 mg/kg/day.

	10 mg/kg/day			200 mg/kg/day		
	Nonpregnant	Pregnant	*P*-value	Nonpregnant	Pregnant	*P*-value
C_{max} (µg/mL)	26.0±14.1	18.5±4.4	0.971	110.1±28.2	120.7±11.1	0.200
AUC_{ss} (µg×h/mL)	259±89	262±103	0.386	1618±407	2057±285	0.579
T_{max} (h)	2.7±0.6	4.3±3.2	0.465	3.7±0.6	8.8±0.0	0.006

Pritelivir was quantified in the plasma of female CD1-IGS mice following oral administration of multiple doses of 10 mg/kg/day and 200 mg/kg/day of pritelivir. The plasma concentration-time profiles of pritelivir are shown in Figs 3 and S1. The PK parameters were determined using Kinetica software (v. 5.1). The data presented as mean±S.D. of three experiments. Statistical analysis was carried out using SPSS software (v28), *t*-test was used for comparisons of pregnant and nonpregnant groups treated with the same dose of the drug.

AUC_{ss}, area under the concentration-time curve at steady state

C_{max}, maximum plasma concentration

T_{max}, time of C_{max}

Table S7. Pharmacokinetic parameters of pritelivir in the tissues of pregnant and nonpregnant female CD1-IGS mice following treatment with the dose of 10 mg/kg/day.

**Tissue**	**Pregnant**			**Nonpregnant**		
	C_{max} (μg/g)	AUC_{ss} (μg×h/g)	AUC_{ss} tissue/ AUC_{ss} plasma ratio	C_{max} (μg/g)	AUC_{ss} (μg×h/g)	AUC_{ss} tissue/ AUC_{ss} plasma ratio
**Brain**	6.1±1.7	67.8±15.3	0.30±0.18	4.72±0.76	56.68±9.70	0.25±0.13
**Liver**	21.1±5.0	257.4±56.0	1.17±0.77	15.85±3.75	163.64±35.71	0.66±0.13
**Lung**	22.6±13.5	282.6±117.2	1.30±1.03	16.51±3.30	183.13±57.21	0.74±0.23
**Heart**	16.0±5.8	188.1±43.8	0.86±0.58	10.52±1.51	116.02±35.55	0.46±0.08
**Kidney**	14.5±4.4	195.2±98.9	0.93±0.83	13.79±5.84	112.39±21.36	0.45±0.08
**Spleen**	15.1±9.8	167.1±165.0	0.92±1.19	7.23±4.23	62.27±22.34	0.26±0.10

Data presented as mean±S.D. of n=3 experiments in pregnant and nonpregnant female mice (each experiment utilized multiple animals, n=1-3 mice per predetermined time point)

AUC_{ss}, area under the concentration-time curve at steady state

C_{max}, maximum tissue concentration

Table S8. Pharmacokinetic parameters of pritelivir in the tissues of pregnant and nonpregnant female CD1-IGS mice following treatment with the dose of 200 mg/kg/day.

Tissue	Pregnant			Nonpregnant		
	C_{max} (µg/g)	AUC_{ss} (µg×h/g)	AUC_{ss} tissue/ AUC_{ss} plasma ratio	C_{max} (µg/g)	AUC_{ss} (µg×h/g)	AUC_{ss} tissue/ AUC_{ss} plasma ratio
**Brain**	33.8±5.86	494±217	0.23±0.07	29.6±4.13	376±49.3	0.24±0.05
**Liver**	156±49.7	2373±490	1.16±0.25	125±14.3	1689±174	1.07±0.15
**Lung**	90.1±5.60	1696±371	0.82±0.07	92.7±15.1	1305±300	0.81±0.05
**Heart**	80.4±7.60	1416±181	0.69±0.03	111±27.3	1220.25±334	0.75±0.02
**Kidney**	82.8±8.27	1344±319	0.65±0.07	80.2±21.6	1107±210	0.69±0.08
**Spleen**	60.4±9.36	872±253	0.43±0.14	47.6±7.46	672±143	0.43±0.12

Data presented as mean±S.D. of n=3 experiments in pregnant and nonpregnant female mice (each experiment utilized multiple animals, n=1-3 mice per predetermined time point)

AUC_{ss}, area under the concentration-time curve at steady state

C_{max}, maximum tissue concentration

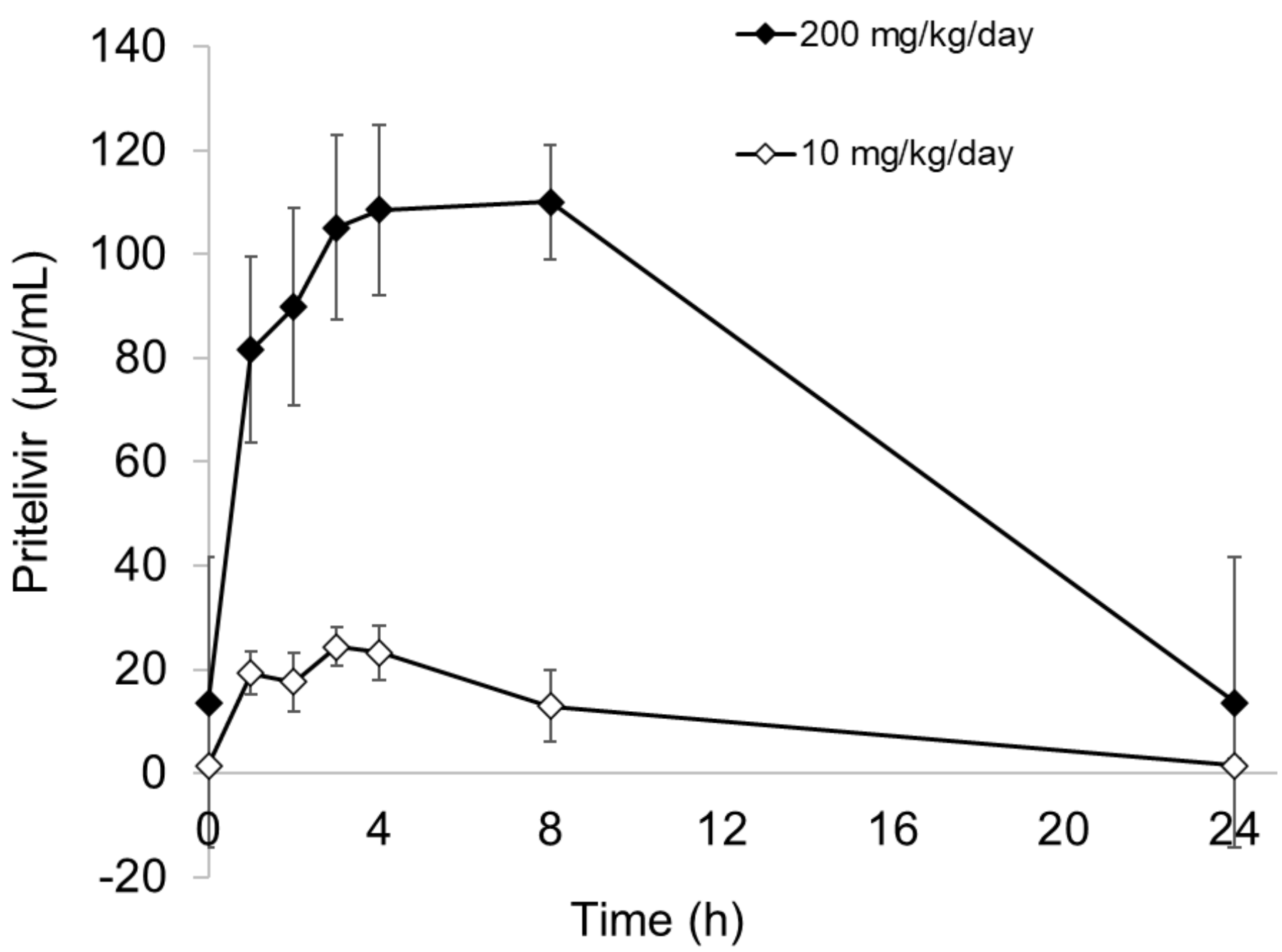

Figure S1. Plasma concentration-time profiles for pritelivir administered to nonpregnant female CD1-IGS mice via gavage at dose levels of 10 and 200 mg/kg/day from gestational day 9 through 13. Animals were sacrificed by CO_2 asphyxiation, followed by bilateral thoracotomy, at the predetermined time points of 1, 2, 3, 4, 8, and 24 h post-dose, and blood samples were obtained by cardiac puncture. Pritelivir in plasma samples was quantified using LC-MS/MS. Data are presented as the mean±S.D. of three separate experiments that utilized 1-3 animals per time point.

A

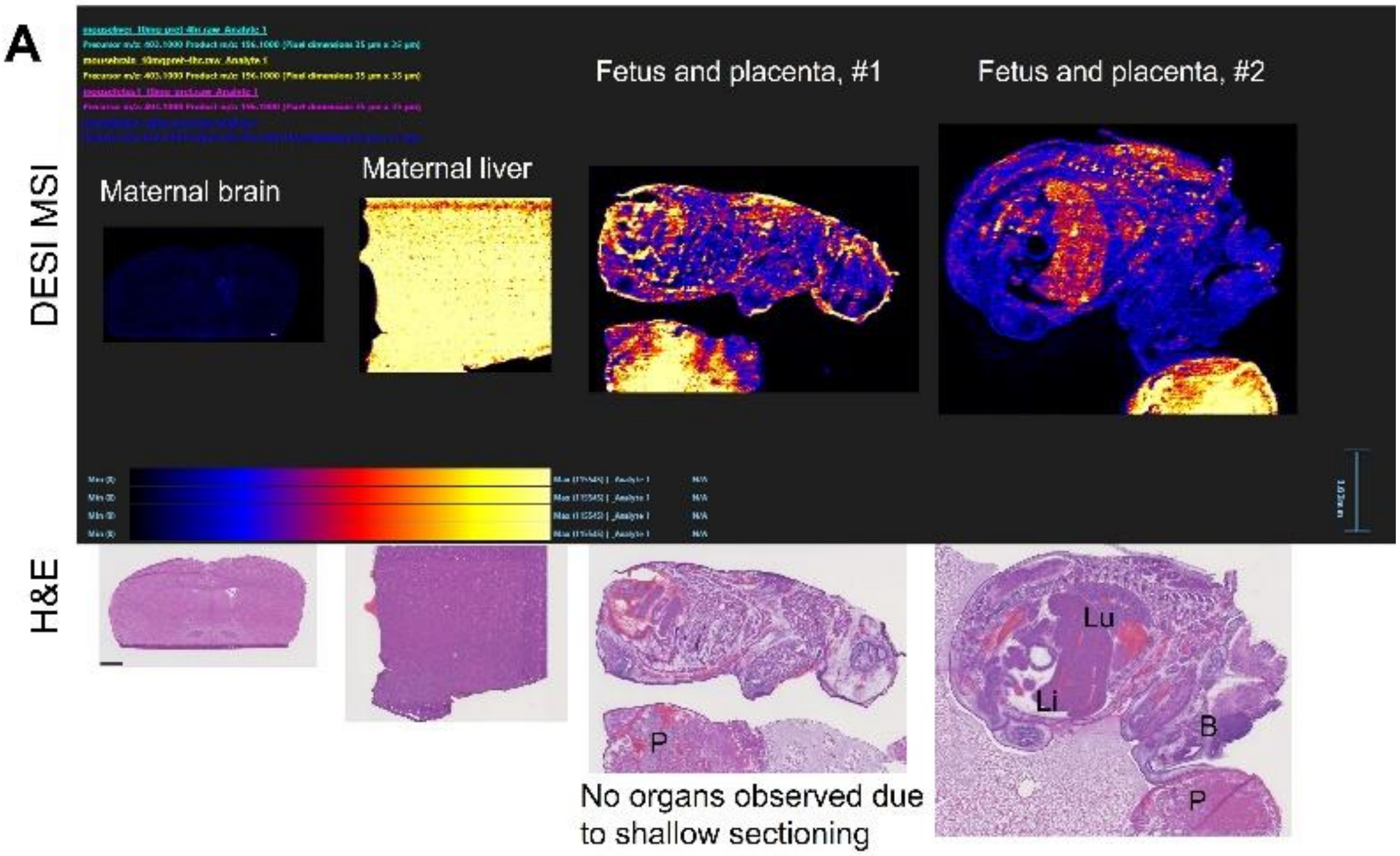
DESI MSI
Maternal brain
Maternal liver
Fetus and placenta, #1
Fetus and placenta, #2
H&E
Lu
Li
B
P
No organs observed due to shallow sectioning

B

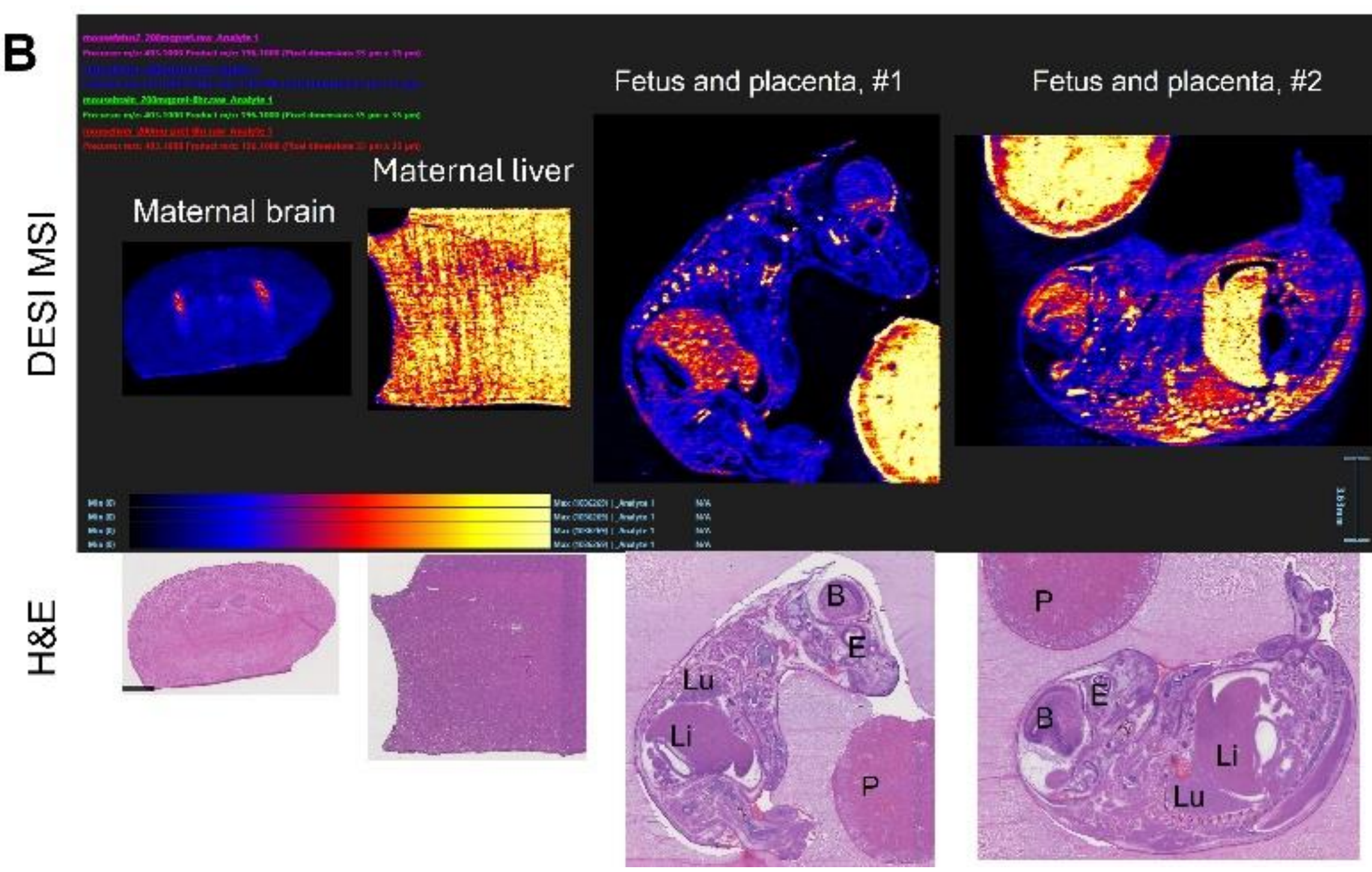
DESI MSI
Maternal brain
Maternal liver
Fetus and placenta, #1
Fetus and placenta, #2
H&E
B
E
Lu
Li
P

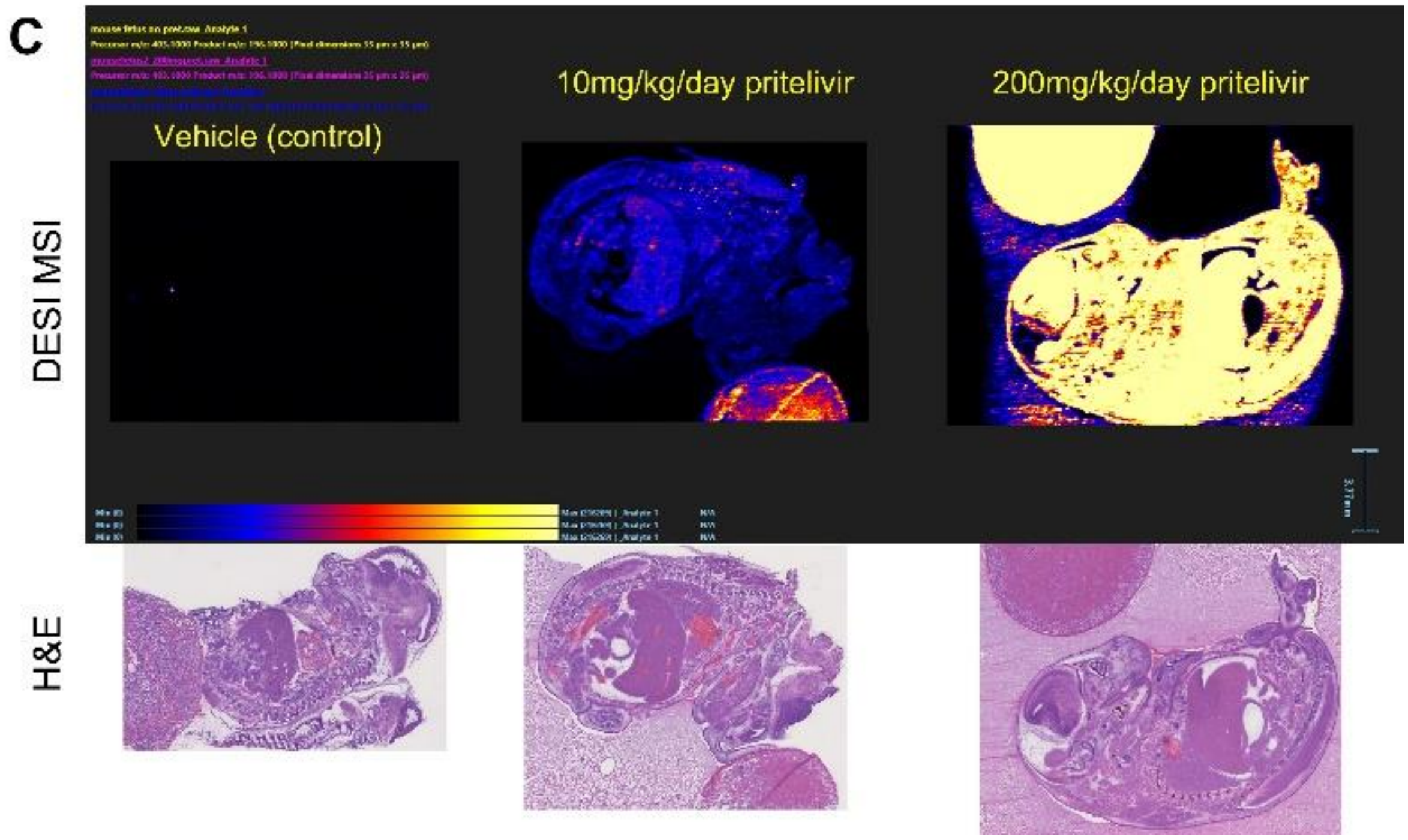

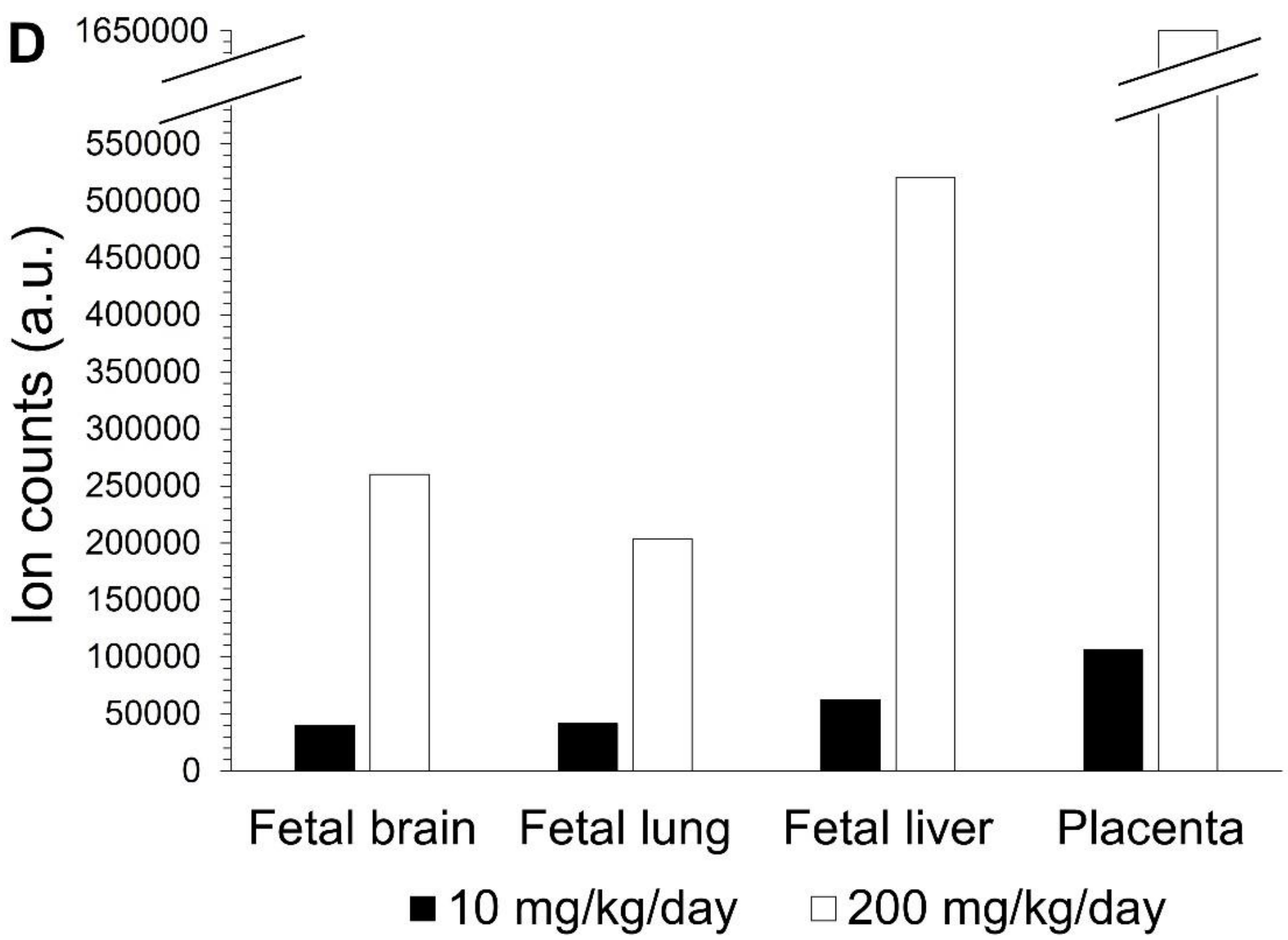

FIGURE S2. Visualization of pritelivir (m/z 491→196) in CD1-IGS mouse maternal and fetal tissues by targeted DESI-MSI following treatment of pregnant mice with 10 mg/kg/day (A) and

200 mg/kg/day (B) from gestational day 9 through 13. Panel C shows representative fetal and placental tissues from animals treated with vehicle (control) and pritelivir (both drug doses) on the same intensity scale (with saturation at the 200 mg/kg/day dose). Panel D shows mean ion signal detected within the selected tissues of interest for representative animals from the 10 mg/kg/day and 200 mg/kg/day dose groups. Maternal animals were sacrificed by CO_2 asphyxiation, followed by bilateral thoracotomy, at 4 h post dose for vehicle-treated and 10 mg/kg/day pritelivir and 8 h post dose for 200 mg/kg/day pritelivir. MSI data was acquired at 40 mm pixel size. DESI-MS signal intensities were normalized to pritelivir-d_4 internal standard spiked into the DESI spray solvent, and MS images are presented scaled individually for each dose (panels A and B) and on the same intensity scale (panel C). Scalebar = 4 mm. B, brain; E, eye; Li, liver; Lu, lung; P, placenta; H&E, Hematoxylin and Eosin; a.u.; arbitrary units.
